# Short versus standard-length implants: A systematic review on clinical outcomes and success rates

**DOI:** 10.6026/973206300212235

**Published:** 2025-07-31

**Authors:** Piyush Javiya, Mitra Esmaili, Jay Soni, Neelam Chandwani, Neelima Chowdary Cherukumalli Kapalavayi, Aishwarya G. Arya, Rashmi Laddha

**Affiliations:** 1 Department of Prosthodontics, Crown & Bridge, K. M. Shah Dental College & Hospital, Sumandeep Vidyapeeth Deemed to be University, Piparia, Waghodia, Vadodara, Gujarat, India; 2 Department of Cariology and Comprehensive Care, NYU College of Dentistry, New York, USA; 3 Department of Dentistry, All India Institute of Medical Sciences, Nagpur, India; 4 Department of Managing Dentist, Brident Dental and Orthodontics Institute, Texas, USA; 5 Deparment of Periodontology and Implantology, KNRUHS Institute, Mallareddy Institute of Dental Sciences, Telangana, India; 6 Department of Periodontology, Dr. RR Kambe Dental College and Hospital, Akola, India

**Keywords:** Short implants, standard-length implants, dental implants, implant success, implant survival, marginal bone loss, clinical outcomes

## Abstract

Dental implants have become a reliable solution for edentulism, yet bone limitations often necessitate alternative approaches such as short implants. This systematic review evaluates and compares the clinical outcomes and success rates of short dental implants (≤8 mm) versus standard-length implants (>8 mm) in various clinical scenarios. Data from multiple studies were analyzed to assess implant survival, marginal bone loss, prosthetic complications and long-term stability. The findings suggest that, when appropriately indicated, short implants demonstrate comparable success and survival rates to their longer counterparts, particularly in atrophic ridges where bone augmentation may be avoided. However, variations in surgical techniques, prosthetic protocols and patient-related factors can influence outcomes. Thus, short implants represent a viable, minimally invasive alternative, provided patient selection and clinical protocols are well-considered.

## Background:

The advent of dental implants has transformed the field of oral rehabilitation, offering patients a reliable and functionally superior alternative for the replacement of missing teeth [1]. Success in implant dentistry is primarily gauged by the long-term osseointegration and clinical performance of the implant, both of which are influenced by numerous patient-related, surgical and prosthetic factors [2]. Traditionally, standard-length implants, defined as those greater than 8 mm in length, have been widely used and extensively validated in clinical practice [3]. However, anatomical limitations such as reduced vertical bone height in the posterior maxilla and mandible often necessitate complex and invasive procedures like bone grafting or sinus augmentation to accommodate these implants [4]. In response to these challenges, short implants typically measuring 8 mm or less have emerged as a minimally invasive alternative that can bypass the need for additional augmentation procedures [5]. Initial skepticism concerning their biomechanical stability and long-term outcome has slowly diminished with advances in implant design, surface treatment and surgical technique [6]. Recent studies more support the fact that short implants, when well-indicated and well-performed, can obtain clinical results comparable to those of their longer counterparts [7]. In spite of the increasing volume of literature favouring short implants, issues still exist concerning their relative performance in survival rates, marginal bone loss, prosthetic complications and appropriateness in different anatomical and loading situations [8]. In addition, differences in study design, implant systems, follow-up periods and patient selection criteria have led to heterogeneous results throughout the literature [9]. Therefore, it is of interest to describe and synthesize current clinical evidence comparing short and conventional-length implants. By evaluating success rates, bone preservation, prosthetic outcomes and patient satisfaction, this review aims to clarify the role of short implants in modern implantology and provide clinicians with an evidence-based framework for treatment planning.

## Clinical rationale for short versus standard-length implants:

The choice of implant length is a key component in dental implant treatment planning and tends to be determined by the volume of bone available at the planned location. In clinical situations wherein the alveolar bone is short - typically seen in the posterior maxilla owing to sinus pneumatization or the mandible as a result of close proximity to the inferior alveolar nerve - placement of normal-length implants often requires sophisticated surgical procedures like sinus floor elevation, vertical ridge augmentation, or nerve lateralization. Although these procedures can reconstruct enough bone volume to support longer implants, they carry additional surgical complexity, morbidity, expense and patient anxiety [10]. Short dental implants provide a good option in such cases by providing an opportunity to place the implant within the native bone anatomy, without the need for extra augmentation procedures. The development of short implants from a failed last-resort to an acceptable primary treatment modality has been motivated by advances in implant macro- and micro-architectures. These are features of better surface texture to enhance osseointegration, increased diameters to expand surface area and altered thread designs that enhance initial stability. Besides, improvement in surgical procedures and immediate loading protocols has broadened the indications for short implants, making them feasible even in those that were earlier considered high-risk [11]. From the patient's perspective, short implants decrease the number of surgeries, reduce healing times and limit post-surgical discomfort, factors that cumulatively lead to higher acceptance and satisfaction. In addition, they are a feasible option in elderly patients or patients with systemic diseases who are not good candidates for extensive grafting. Short implants in edentulous cases, especially in the posterior segments, can make fixed prosthetic solutions possible without infringing on anatomical structures. While short implants are obviously clinically and pragmatically beneficial, successful use is dependent on proper case selection, proper prosthetic planning and knowledge of their mechanical limitations. This requires an intensive comparison with standard-length implants to define the settings in which they can be used as good substitutes without compromising long-term results.

## Biomechanical and biological considerations:

The achievement of dental implants is it short or standard length is largely dependent on the biomechanical loading of functional loads and the adjacent tissues' biological response. Standard length implants have greater bone contact surface area, previously reported to relate to better primary stability as well as long-term outcomes. This, however, has been impugned with the advent of short implants with innovative design features targeted at enhancing their osseointegrative capability and mechanical properties. Biomechanically, short implants possess an increased crown-to-implant (C/I) ratio with the potential to precipitate concerns regarding the focussing of occlusal loads on the crestal bone level. However, numerous studies have established that with favorable implant placement, splinting techniques and occlusal adjustments, such biomechanical disadvantage can be overcome [12]. Models of finite element analysis indicate that stress distribution depends more on prosthetic design and bone quality rather than implant length. Therefore, shorter implants inserted in dense bone and loaded axially are likely to function as well as longer implants under more favorable conditions. From a biological perspective, osseointegration is not just a function of implant length but also of the surface characteristics of the implant, surgical technique and host bone quality. Short implants are now widely manufactured with roughened or conditioned surfaces - i.e., sandblasted, acid-etched, or plasma-sprayed coatings that promote bone-to-implant contact and enhance healing. Moreover, increased diameters in short implants help increase surface area and offer higher resistance to lateral forces. Peri-implant bone remodeling response is yet another important consideration. Clinical evidence indicates that marginal bone loss with short implants is not substantially greater than in usual-length implants, especially when inserted under satisfactory biomechanical conditions. Furthermore, the capacity of short implants to circumvent further surgical trauma might offer biological benefits, since they leave the native bone architecture intact and minimize the potential for graft-related complications or infection [13]. Although short implants present unique biomechanical challenges because of their compromised length, technology in implant geometry and prosthetic management has greatly enhanced their clinical predictability. Short implants have the capability of establishing a stable bone-implant interface and transferring functional loads properly under controlled situations, thereby providing biological and mechanical results that are similar to those of standard-length implants.

## Survival rates and success parameters:

The assessment of implant performance is usually focused on two major endpoints: survival rate and success rate. Implant survival implies the existence of the implant within the oral cavity, independent of surrounding tissue status or prosthetic complications, while success involves more demanding criteria like no pain, no infection, no mobility, peri-implant radiolucency and extensive bone loss. Recent reports have repeatedly demonstrated that short implants can have survival rates equal to those of regular-length implants. Most reports and systematic reviews of clinical findings place survival rates for short implants at 91% to 98%, which is remarkably similar to the survival rates of regular implants, which are usually in the range of 92% to 99%. A significant meta-analysis concluded that with the use of current short implants and proper technique and loading protocol, there is no significant difference in survival statistically with longer implants, even after follow-up durations of 3-5 years [14]. Success rates, while less consistent, likewise indicate encouraging patterns. A number of clinical trials have reported greater than 90% success with short implants with no higher prevalence of peri-implantitis or mechanical failure compared to traditional-length counterparts. Among the crucial factors leading to these results are the host bone quality, surgical technique and prosthetic loading protocol. Significantly, cantilever extensions and splinted restorations tend to be used with short implants to decrease stress concentrations and promote durability. For completely edentulous posterior segments where bone height vertically is deficient, short implants have been found particularly useful by obviating the need for grafting while still providing acceptable long-term stability. In addition, research has pointed out that contemporary short implants, particularly those installed through flapless or minimally invasive surgery, have a propensity to maintain the contours of the soft tissue and ensure esthetically favorable results [15]. These results together indicate that short implants, judiciously applied, are no worse in survival and success rate compared to conventional-length implants. Nevertheless, regular follow-up and properly controlled long-term research are needed to support their validity for extended patient groups and varying conditions.

## Marginal bone loss and prosthetic outcomes:

Marginal bone loss (MBL) is among the most important parameters to assess the long-term success and biological stability of dental implants. Excessive bone loss may jeopardize osseointegration, esthetics and survival of the implant and hence, it is considered an important outcome measure in comparative studies between short and standard-length implants. Previous clinical reservations about short implants stemmed from the supposition that their decreased length would cause adverse stress distribution and ensuing greater marginal bone loss. However, recent evidence disproves this. A number of long-term follow-up studies have revealed that the average marginal bone loss with short implants is no different from that with the conventional-length implant. Indeed, most studies quote bone loss values well within the safe range of 1.5 mm in the first year and <0.2 mm per year subsequently. A systematic review revealed that short implants in non-augmented ridges can even have slightly lower MBL as a result of lesser surgical trauma and preservation of native bone [16]. The application of platform-switching abutments, conical connections and surface-treated implants has also led to enhanced bone preservation in both implant designs. Additionally, bone loss patterns would seem to be more directly determined by surgical technique, occlusal overload and peri-implant soft tissue control than by implant length perse. Flap design, insertion torque and emergence profile are among the important factors influencing peri-implant bone behavior. From a prosthetic perspective, issues with short implants primarily concern their behavior under functional loading. Large crown-to-implant ratios, typical in short implant restorations, were previously considered a mechanical risk factor for component fracture or loosening [17]. Clinical evidence, however, has not shown a clear increase in prosthetic complications including abutment screw loosening, porcelain chipping, or crown dislodgement compared to conventional implants. This is especially the case when occlusal plans are well-planned and Para functional behaviors are controlled. Splinting short implants or integrating them with longer implants within a common prosthetic unit has been demonstrated to distribute occlusal forces more evenly, lessening biomechanical stress. Moreover, advancements in digital workflows and CAD/CAM prosthetics have enhanced accuracy and passive fit, lessening the frequency of prosthetic failure. The marginal bone levels and prosthetic results of short implants are within acceptable clinical ranges and not materially worse than those of regular-length implants. With sound treatment planning and practice, short implants can sustain functionally and esthetically adequate restorations with a similar risk profile.

## Comparative evidence from clinical studies:

Critical review of comparative clinical research constitutes the foundation of assessing the effectiveness of short versus conventional-length implants. During the last decade, a rising number of randomized controlled trials (RCTs), cohort studies and retrospective examinations have examined this topic, offering a stable body of evidence suitable for systematic review. Many RCTs have revealed no statistically relevant differences in survival and success rates between short and standard implants, even if follow-up periods lasted for five years or more. In posterior mandible or maxilla studies, where vertical bone height is restricted, short implants had survival rates equal to those obtained with bone-augmented standard implants. Significantly, these studies also recorded decreases in surgical time, postoperative complications and patient morbidity in the short implant groups owing to bypassing extra grafting procedures [18]. Retrospective investigations have also confirmed the above conclusions, with evidence suggesting that short implants can endure similar loading forces and preserve peri-implant tissue integrity in the long term. Systematic reviews and meta-analyses have produced similar findings that more than 3,000 implants found no clinically significant differences in implant survival rate between short and conventional implants. Additionally, in comorbid patients or older patients, short implants tended to work better because they presented lower surgical risk [19]. It should be mentioned that heterogeneity in prosthetic protocols, patient selection criteria and implant systems between studies can affect reported results. Some studies employed the use of splinted prostheses to enhance mechanical distribution, while others tested single-tooth replacements, where every single tooth replacement has specific biomechanical factors to consider. Heterogeneity in follow-up periods and also in reporting standards is another problem for the absolute conclusion. In spite of these differences, the general trend in literature is highly supportive of the feasibility of short implants as a substitute for longer implants, particularly in compromised anatomical situations. The evidence highlights that with correct surgical technique, good-quality implant design and proper prosthetic planning, short implants can provide comparable clinical performance with less invasiveness.

## Patient-centered outcomes and indications:

In modern implantology, treatment outcome evaluation goes beyond clinical survival to encompass patient-oriented parameters like quality of life, functional satisfaction, esthetics and general treatment experience. The option between short and regular-length implants has major impacts in all these aspects, particularly taking into consideration the surgical invasiveness, treatment time and related morbidity. Short implants provide a less invasive technique, which is especially ideal for patients with systemic health complications, old age, or increased surgical risk. Without the complicated augmentation procedures like sinus lift or vertical ridge augmentation, short implants minimize postoperative pain, operating time and surgical sessions. These benefits tend to equate to increased patient acceptance and adherence, particularly among those fearful of invasive procedures or extended recovery times [20]. Functional results, such as chewing efficiency and speech, have been found to be the same for short and normal-length implant restorations. Investigations comparing patient satisfaction scores and oral health-related quality of life (OHRQoL) have invariably revealed positive outcomes in both groups. Indeed, patients with short implants tend to report higher satisfaction because of the ease and expediency of treatment, less pain and quicker return to normal function. Aesthetic results, especially in the posterior areas where short implants are most often inserted, are usually acceptable. Although anterior uses are less common because of spatial constraints and esthetic considerations, stringent case selection and prosthetic design can provide acceptable results even here. From an indication perspective, short implants are well suited for posterior edentulous areas with vertical bone restrictions, medically compromised patients where grafting is contraindicated and those requiring accelerated treatment protocols. They are also beneficial in geriatric populations where bone volume is naturally reduced and tolerance for invasive surgical procedures is low [21]. But optimal use of short implants continues to require diligent clinical discretion. Bone quality, occlusal plan, parafunctional habits and systemic health all need to be carefully evaluated to decide on suitability. Short implants may also be contraindicated in situations with widespread cantilevers, unsupported long-span prostheses, or in the presence of extreme bone atrophy where primary stability cannot be ensured. Generally speaking, short implants represent a patient-friendly option that is well within the paradigm of current ideals for minimally invasive dentistry and patient-centered dentistry. With their capacity to achieve functional and esthetic results with decreased morbidity, they are an important treatment option in carefully chosen cases.

## Current challenges and considerations:

Even with the growing popularity of short implants as a practical alternative to conventional-length implants, there remain specific clinical and technical issues that affect their use and long-term results. A critical appraisal of these limitations is needed in order to harness the advantages while minimizing the potential risks involved. One of the primary concerns is the biomechanical limitation caused by a smaller implant-bone interface that may compromise load distribution, particularly under oblique or lateral forces. Despite improvements in macro-geometry and surface treatments of implants that have greatly improved osseointegration and stability, short implants are still mechanically less tolerant in scenarios of compromised bone quality or elevated occlusal stress. This requires careful occlusal planning and the potential use of auxiliary approaches like implant splinting to minimize the dangers of overloading [22]. Technique also plays an important role. Gaining primary stability using short implants may be technique-dependent, especially in the maxilla, where cancellous bone is prevalent. The significance of atraumatic insertion, proper angulation and optimal insertion torque cannot be emphasized enough. Even minor variations in these values may jeopardize the prognosis of short implants, especially in single-tooth restorations or immediately loaded cases. Another issue that persists is the heterogeneity in study design and outcome reporting throughout the literature. The characterization of "short" implant differs between studies, with some classifying it as ≤8 mm and others as implants ≤10 mm. In addition, heterogeneity introduced by variability in follow-up periods, patient inclusion criteria and prosthetic strategies reduces the direct comparability of outcomes. This point to the necessity for more standardized research designs and more uniform outcome measures to enhance the evidence base [23]. Moreover, although short implants have shown promising results in controlled clinical settings, their performance in day-to-day, general practice conditions has yet to be adequately tested. Factors like clinician experience, patient compliance and inconsistency in prosthetic loading protocols might influence the reproducibility of outcomes in routine clinical practice. Lastly, economic factors, while generally favorable owing to the avoidance of grafting procedures, would also need to consider the possible expense of prosthetic complications or retreatment in case of failure. These issues need to be balanced by clinicians when explaining treatment options to patients and making evidence-based recommendations. Thus, although short implants have many benefits, their clinical application must be informed by a deep understanding of their mechanical loading, biological integration and procedural needs. Further development towards standardized clinical methodologies, practitioner education and patient-specific risk assessment is crucial to their optimization of long-term success across various implant situations. Sensitivity of their limitations and adherence to evidence-based practice are necessary to maximize outcomes and develop wider applications of short implants in contemporary implantology.

## Discussion:

The transformation of short dental implants from a previously doubting alternative to a commonly accepted clinical option represents a dramatic change in contemporary implantology. The present review emphasizes the expanding evidence base for the similar clinical performance of short implants compared with conventional-length implants, especially when analyzed in terms of crucial endpoints including survival rates, marginal bone level maintenance, prosthetic success and patient satisfaction. Short implant survival rates cited in recent literature continue to come close to those of longer counterparts. The equivalence in performance is largely credited to advances in implant surface technologies, macro design and surgical methods, all serving to improve biomechanical stability and biological integration in shorter fixtures. Of particular note, research involving wide-diameter short implants or platform-switching abutments has yielded especially positive results, which points to the criticality of prosthetically guided planning and system choice [24]. Marginal bone loss, long the bane of short implants, has not proven to be clinically significantly increased relative to full-length implants as long as prosthetic loads are meticulously controlled. Actually, short implants with no associated bone grafting procedures are generally found in a number of scenarios to better retain crestal bone because there is less surgical trauma and there is native bone structure preserved. These results follow minimally invasive dentistry trends where biological retention takes precedence over traumatic intervention. The second relevant factor to be identified from the examined literature is short implants' high degree of patient-centered satisfaction. The ability not to use grafting, decrease treatment durations and eliminate postoperative complications greatly adds to successful patient-reported outcomes. For frail older adults or individuals with systemic constraints, short implants present a safer and more effective route to oral rehabilitation [25]. Yet, it is also evident that the success of short implants is highly case and clinically dependent. Unfavourable bone quality, uncontrolled occlusal forces and poor surgical technique continue to be potential risk factors for failure. The absence of broadly accepted definitions of "short" implants and the variability in methodologies employed in studies complicate comparisons and extrapolations. Moreover, although most of the included studies present outcomes between 1 and 5 years, long-term follow-up after more than a decade is still limited. A comparison between this review and previous analyses shows a general trend of confirmation of short implants, especially in posterior areas with reduced vertical bone height. However, the use of short implants should not be allowed to take precedence over the use of standard-length implants in situations where patient anatomy and bone volume are good, or where long-span prostheses require increased anchorage. Finally, the clinical choice between short and standard-length implants should be dictated by patient needs, anatomical limitations and restorative requirements. Complete knowledge about the biomechanics and biological behavior of implants of different lengths and an understanding of patient wishes and expectations will help in achieving predictable and successful results.

## Conclusion:

The clinical evidence supports short dental implants as a predictable alternative to standard‑length implants in cases where anatomical limitations preclude longer fixtures without augmentation. Survival and success rates are comparable when appropriate case selection, surgical technique and prosthetic planning are employed. Short implants offer reduced morbidity, treatment time and cost, aligning with minimally invasive, patient‑centered practice provided biomechanical and biological considerations are carefully managed.
